# Bone angiocrine factors

**DOI:** 10.3389/fcell.2023.1244372

**Published:** 2023-08-03

**Authors:** Domenico Ribatti, Antonio d’Amati

**Affiliations:** Department of Translational Biomedicine and Neurosciences, University of Bari Medical School, Bari, Italy

**Keywords:** angiocrine factors, bone vascularization, FGF, HIF1-α, Notch, VEGF

## Abstract

Angiogenesis in the bone is unique and involves distinctive signals. Whether they are created through intramembranous ossification or endochondral ossification, bones are highly vascularized tissues. Long bones undergo a sequence of processes known as endochondral osteogenesis. Angiogenesis occurs during the creation of endochondral bone and is mediated by a variety of cells and factors. An initially avascular cartilage template is invaded by blood vessels from the nearby subchondral bone thanks to the secreted angiogenic chemicals by hypertrophic chondrocytes. Vascular endothelial growth factor (VEGF), one of several angiogenic molecules, is a significant regulator of blood vessel invasion, cartilage remodeling, and ossification of freshly created bone matrix; chondrocyte proliferation and hypertrophy are facilitated by the production of VEGFA and VEGF receptor-2 (VEGFR-2), which is stimulated by fibroblast growth factors (FGFs). NOTCH signaling controls blood capillaries formation during bone maturation and regeneration, while hypoxia-inducible factor 1 alpha (HIF1-a) promotes chondrocyte development by switching to anaerobic metabolism. To control skeletal remodeling and repair, osteogenic cells release angiogenic factors, whereas endothelial cells secrete angiocrine factors. One of the better instances of functional blood vessels specialization for certain organs is the skeletal system. A subpopulation of capillary endothelial cells in the bone regulate the activity of osteoprogenitor cells, which in turn affects bone formation during development and adult homeostasis. Angiogenesis and osteogenesis are strictly connected, and their crosstalk is essential to guarantee bone formation and to maintain bone homeostasis. Additionally, pathological processes including inflammation, cancer, and aging include both bone endothelial cells and angiocrine factors. Therefore, the study and understanding of these mechanisms is fundamental, because molecules and factors involved may represent key targets for novel and advanced therapies.

## 1 Introduction

Whether they are created through intramembranous ossification or endochondral ossification, bones are highly vascularized tissues. Long bones undergo a sequence of processes known as endochondral ossification that depend on the interaction between chondrogenesis, the synthesis and resorption of cartilage, and bone formation (osteogenesis). When osteogenesis begins and the primary ossification center is formed during endochondral osteogenesis, signals sent by hypertrophic chondrocytes cause blood vessels to invade an initially avascular cartilage template. Like how postnatal vascular ingrowth into hypertrophic cartilage of the long bone’s distal ends causes secondary ossification centers to arise. Postnatal growth and bone length are accompanied by the emergence of morphologically and molecularly different capillary subpopulations (Owen-Woods and Kusumbe, 2022).

## 2 The role of VEGF in promoting endochondral ossification

Hypertrophic chondrocytes secrete angiogenic molecules, such as vascular endothelial growth factor (VEGF), fibroblast growth factor-2 (FGF-2), transferrin, and matrix metalloproteinase-9 (MMP-9), which draw blood vessels from the nearby subchondral bone during the creation of endochondral bone. A VEGF gradient that regulates sprouting angiogenesis is produced when three VEGF isoforms (VEGF120, VEGF164, and VEGF 188) secrete themselves together (Baron et al., 1994; Carlevaro et al., 1997; Vu et al., 1998; HORNER et al., 1999; Carlevaro et al., 2000).

MMP-9 causes the extracellular matrix to release angiogenic factors. In homozygous mice with a null mutation in the MMP-9 gene, an increase of the hypertrophic chondrocyte area similar to that reported in VEGF-defective animals can be seen (Vu et al., 1998).

Invasion of blood vessels into the epiphyseal growth plate, remodeling of hypertrophic cartilage, and osteoblasts’ ossification of newly created bone matrix (trabeculae) are all significantly regulated by VEGF (Gerber et al., 1999; Gerber and Ferrara, 2000). VEGFA is released by differentiated osteoblasts (Kusumbe et al., 2014). The differentiation of osteoblasts and osteoclasts is stimulated by VEGF secreted by osteoblasts, encouraging bone remodeling and repair (Berendsen and Olsen, 2014; Hu and Olsen, 2016). Osteoclasts express VEGF receptors (VEGFRs), and VEGF promotes their survival and bone resorption (Nakagawa et al., 2000).

Blood vessel invasion of the epiphyseal growth plate is almost eliminated by intravenously injecting soluble VEGFR proteins that block VEGF. Due to the poor production of apoptotic signals to hypertrophic chondrocytes and the unsuccessful recruitment of chondroblasts and osteoblasts, impaired blood vessel development is to blame for impaired trabecular bone formation. A 7 to 10-fold enlargement of the hypertrophic chondrocyte zone and a decrease in longitudinal bone lengthening occur after defective remodeling of this region (Gerber et al., 1999). Withholding anti-VEGF therapy entirely undoes all the results. The primary ossification center of animals expressing the VEGF-A120 isoform of VEGFA, an isoform of VEGFA that does not bind heparin sulfate, showed altered cartilage differentiation marker expression levels and a delay in vascular invasion (Maes et al., 2002; Zelzer et al., 2002). Furthermore, terminally differentiated chondrocyte buildup in the growth plates and a delay in vascular invasion of the major ossification centers are seen in VEGFA-conditional knock-out animals (Zelzer et al., 2004). Endothelial cells encourage the terminal differentiation of resting chondrocytes into hypertrophic cells in addition to encouraging the apoptosis of hypertrophic chondrocytes (Bittner et al., 1998).

## 3 Other factors involved in angiogenesis during endochondral ossification

The DDL4-NOTCH system is required for blood capillary development during bone maturation and regeneration (Ramasamy et al., 2014). Endothelial cell proliferation and the development of type H vessels in bone are encouraged by NOTCH signaling in bone endothelial cells, which is regulated by regional blood flow. Endothelial cells produce signals during NOTCH activation that are necessary for chondrocyte maturation, Sox9 expression, and VEGF production (Ramasamy et al., 2014). The amount of secreted Noggin is decreased by endothelial cell NOTCH loss, which inhibits bone morphogenic proteins (BMPs) and promotes bone development. Bone development is hampered by endothelial cell-specific ablation of ADAM-10, an MMP facilitating Notch signaling activation (Glomski et al., 2011). DDL4-NOTCH is only found in capillaries of type H. Mice with endothelial cell-specific loss of function exhibit decreased VEGF expression as well as type H capillary loss (Ramasamy et al., 2014).

Chondrocyte proliferation and hypertrophy are facilitated by the production of VEGFA and VEGFR-2, which is stimulated by FGFs (Seghezzi et al., 1998). In addition, the absence of FGF-9 and FGF-19 decreases the amount of hypertrophic chondrocytes (Montero et al., 2000), slows chondrocyte proliferation, delays skeletal vascularization, and decreases osteoblast/osteoclast recruitment to the growth plate in mice (Hung et al., 2007; Liu et al., 2007).

**FIGURE 1:**
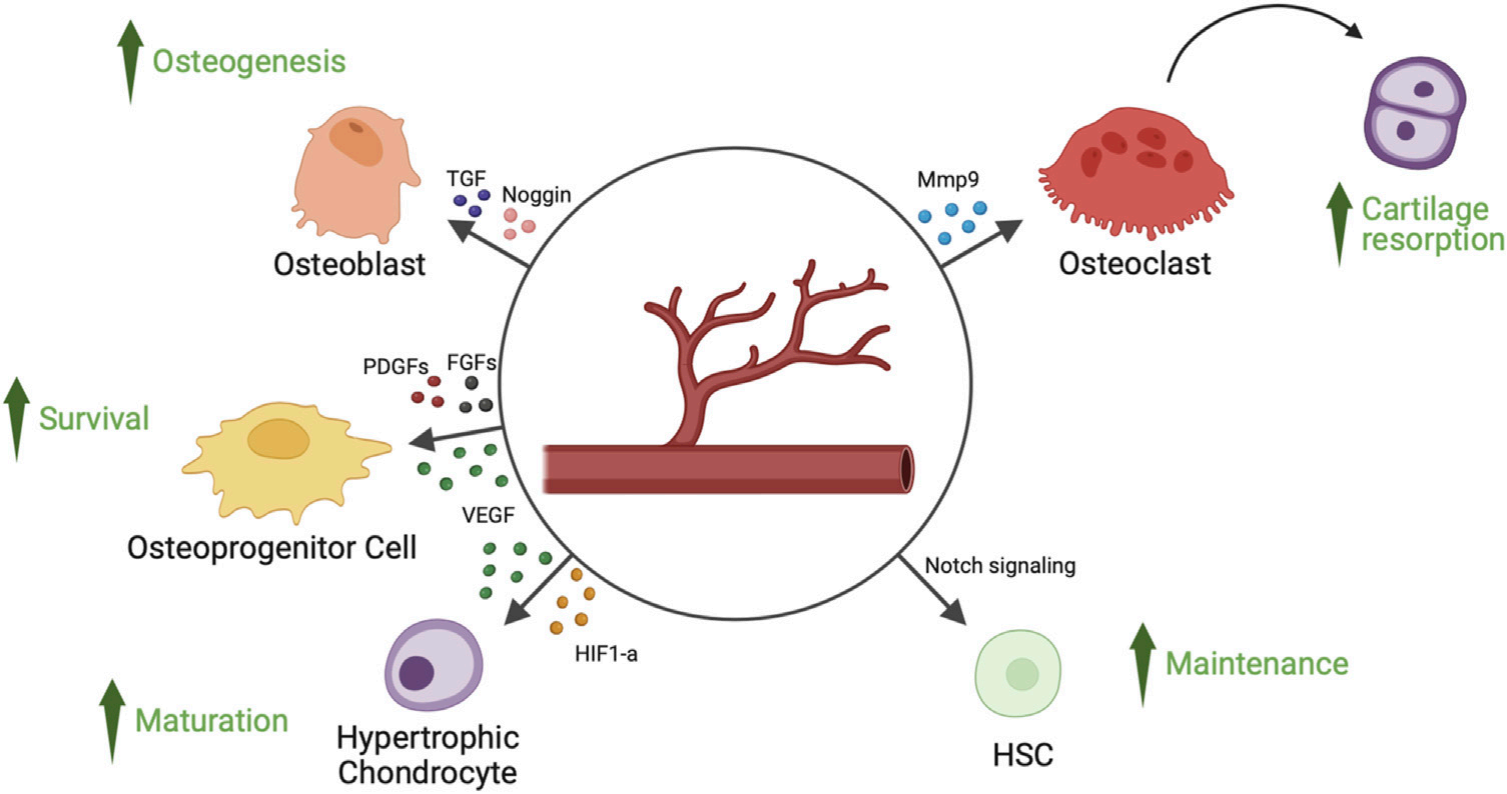
Angiocrine factors released by endothelial cells of bone’s blood vessels and their target cell types. Endothelial cells of bone’s blood vessels secrete several angiocrine factors, capable of regulating hypertrophic chondrocyte maturation, osteoprogenitor cells survival, osteoblast-mediated osteogenesis, osteoclast-mediated cartilage resorption and HSCs (hematopoietic stem cells) survival. FGFs, fibroblast-derived growth factors; HIF1-a, hypoxia inducible factor 1-alpha; Mmp9, matrix metalloproteinase 9; PDGFs, platelet-derived growth factors; TGF, transforming growth factor; VEGF, vascular endothelial growth factor.

Hypoxia inducible factor 1 alpha (HIF1-α) promotes chondrocyte development by using an anaerobic metabolism (Bentovim et al., 2012). Chondrogenesis and osteogenesis are impacted by the absence of HIF1-α because extracellular matrix secretion is decreased (Saito et al., 2010; Yang et al., 2010; Bentovim et al., 2012). Reduction in bone volume and vascularity is linked to HIF1-α deletion in osteoblasts (Schipani et al., 2001). Otherwise, increased angiogenesis and osteogenesis are linked to overexpression of HIF1-α (Yang et al., 2010). While endothelial cell-specific deletion of Von Hipper Lindau gene, which stabilizes endothelial HIF1-α, results in an increase in type H capillary-dependent angiogenesis and osteogenesis, endothelial cell-specific inactivation of HIF1-α is the cause of a reduction in the number of type H vessels and of reduced osteogenesis (Figure 1) (Kusumbe et al., 2014).

## 4 Angiocrine role of endothelial cells during endochondral ossification

The skeletal system is evidence of the functional organ-specific specialization of blood vessels, where a subset of capillary endothelial cells regulates osteoprogenitor cell (OPC) behavior and, consequently, the creation of bone during development, adult homeostasis, and aging.

Perivascular osteoprogenitor cells are supported in their growth and biological functions by specialized CD31hi Emcnhi (type H) capillaries (Kusumbe et al., 2014). The sialoglycoprotein endomucin and CD31 are highly expressed in type H capillaries, which characterizes them. Furthermore, compared to type L vessels, type H vessels have higher blood flow and amounts of oxygen and nutrients (Ramasamy, 2017). Blood flow is essential for the angiogenesis of bone and the development of type H capillaries (Ramasamy et al., 2016). The reduction of blood flow to bone decreases the number of type H capillaries, leading to a reduction of OPCs and new bone formation (Ramasamy et al., 2016). The metaphysis, the area around the growth plate, and the periosteal and endosteal surfaces of the diaphysis all contain type H vessels. These vessels coordinate osteoblast and osteoclast intercellular signaling and produce substances that promote the growth and development of OPCs (Peng et al., 2020). OPCs are selectively located close to type H capillaries and are detected by the expression of the transcription factor Osterix; however, they are not present close to type L vessels. Osteogenic factors are secreted by type H endothelial cells, which help keep Osterix^+^ OPCs alive (Kusumbe et al., 2014). Stem cell factor (SCF) secreted by type H capillaries is another angiocrine factor involved in hematopoietic stem cell (HSC) maintenance (Comazzetto et al., 2019). Endothelial NOTCH activation, which promotes expansion of type H capillaries (Ramasamy et al., 2014), is responsible of an increase of platelet derived growth factor receptor beta (PDGFR-β)/Nestin/Neuron Glial Antigen-2 (NGA-2)^+^ perivascular cells, HSCs, and SCF levels (Szade et al., 2018).

The formation of type H capillaries and subsequent osteogenesis may be aided by PDGF, which is supplied by osteoclast progenitor cells (Xie et al., 2014), and, in close proximity to the development plate, a subpopulation of vessel-associated osteoclasts encourages the anastomosis of distal type H vessels (Romeo et al., 2019). This new subset of osteoclast is specifically associated with type H vessels at the growth plate, acting as vessel-associated osteoclasts, distinct from bone-associated osteoclasts. Vessel-associated osteoclasts are not involved in resorbing bone matrix, but favor type H vessel functional activities (Romeo et al., 2019). Transition vessels connect type H capillaries to the diaphysis’ highly branching sinusoidal vessels, which are encircled by hematopoietic cells (Ramasamy et al., 2016). Age-related reductions in OPCs and bone mass are also accompanied by a decrease in the number of type H capillaries (Kusumbe et al., 2016).

**FIGURE 2:**
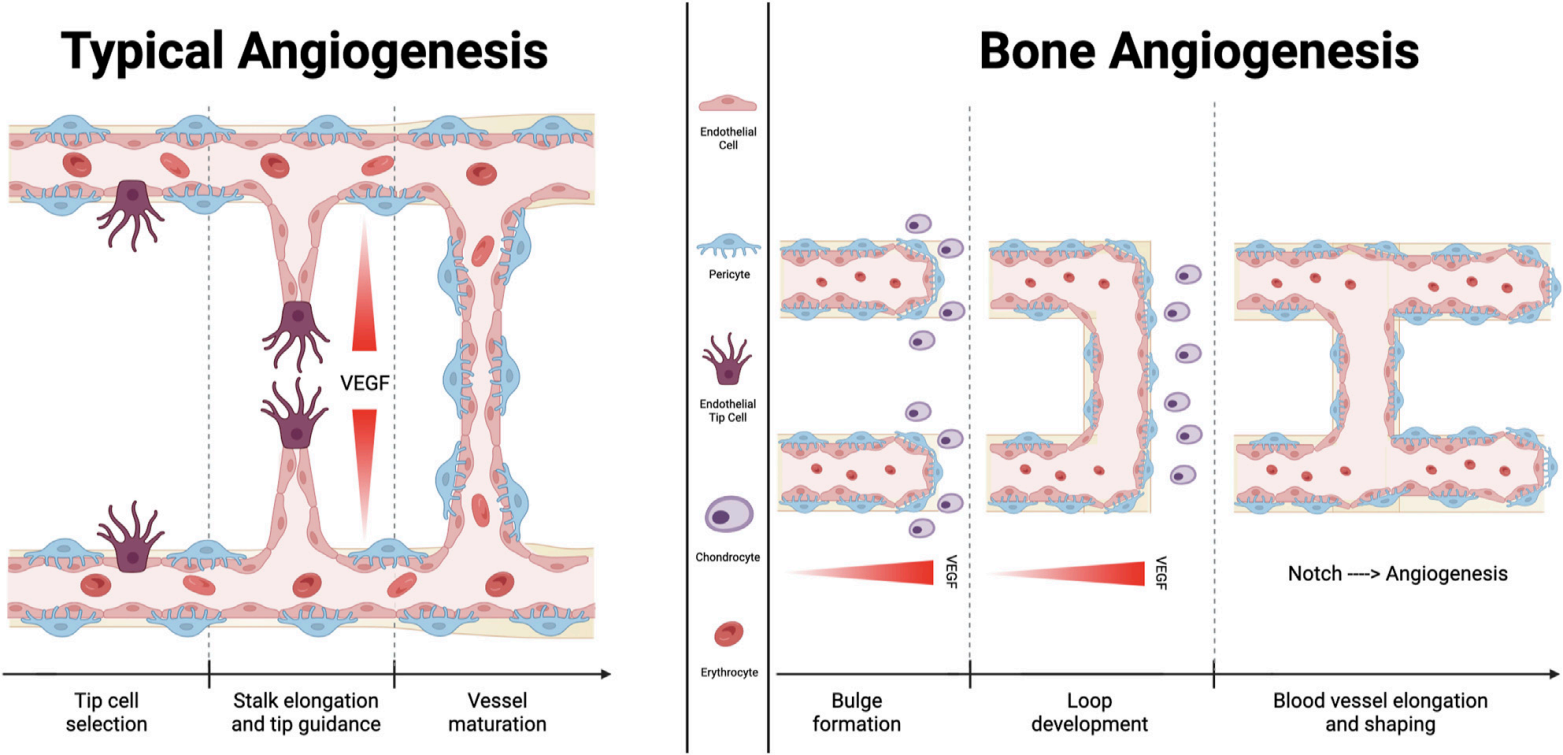
Comparison between typical angiogenesis and bone angiogenesis. Angiogenesis is essential for sustaining the microvasculature and for fetal development. Fundamentally, classical angiogenesis requires the creation of a tip cell, which is followed by the elongation of a stalk that extends following the VEGF gradient of concentration. Bone angiogenetic process, which is described as vessel bulging, has been specifically documented only in the bone tissue. It has been demonstrated that the signaling molecule “Notch” functions differently in this subtype of angiogenesis than it does in other tissues, supporting endothelial cells proliferation and vessel expansion. Additionally, chondrocytes promote the development of new blood vessels by secreting several molecules with angiocrine effects.

Vascular buds that are immediately adjacent to the growth plate, metaphyseal vessel columns, and endosteal capillaries are examples of type H endothelial cells, whereas sinusoidal CD31 low Emcnlow (type L) endothelial cells are present in the bone marrow. Sca-1low and VEGFR3^+^ are characteristics of type L endothelial cells (Kunisaki et al., 2013), which are primarily sinusoidal-like vessels (Langen et al., 2017) that drain into the central vein (Kusumbe et al., 2014). Type H vascular buds can invade the growth plate by resorbing hypertrophic chondrocytes, which are produced by distal vessel arches (Kusumbe et al., 2014). Contiguous buds that have undergone anastomotic fusion create new, arch-shaped vessels that can later give rise to other buds (Figure 2).

Osteoclasts, bone mesenchymal stromal cells (BMSCs), and OPCs are linked to metaphyseal type H vasculature. The bone marrow cavity at the transition zone expands as the metaphysis shrinks following the cessation of embryonic growth. Hematopoietic and reticular cells can be found in bone marrow, but immature BMSCs are primarily found in the metaphysis and endosteum. Endothelial sprouting and remodeling of type H vascular columns into sinusoidal type L vasculature, as well as osteoclast removal of trabecular bone at the metaphyseal-diaphyseal interface, are all components of bone marrow growth (Tuckermann and Adams, 2021).

Embryonic long bones are rich in type E endothelial cells, which can develop into type H cells, which can then produce type L sinusoidal endothelial cells as well as venous and arterial endothelial cells. Although sinusoidal veins are directly connected to the major central vein, it is still unknown how venous endothelial cells differ from other endothelial cell groups in terms of lineage (Tuckermann and Adams, 2021).

## 5 Therapeutic perspectives

Type H capillaries act as biomarker for osteoporosis and bone loss in humans (Wang et al., 2017). In osteoporosis, there is a decrease in type H capillaries, and an increase of type H vessels may be associated with an increase in bone formation through an increase in OPCs (Xie et al., 2014). In aged individuals, the number of type H vessels is reduced, consistent with an increased risk to develop osteoporosis (Wang et al., 2017).

Type H endothelium is a potential therapeutic target to regenerate vascularized bone in different pathological conditions, including osteoporosis, aging, and fracture healing. In this context, systemic treatment with recombinant SLIT3, which promotes type H endothelium growth, ameliorates bone loss in experimental models of fracture healing and postmenopausal osteoporosis (Xu et al., 2018). Moreover, growth of type H endothelium can be promoted by the activation of NOTCH signaling and stimulation of osteoblastic function through endothelial secretion of the BMP inhibitor Noggin (Ramasamy et al., 2014).

Halofuginone, a specific inhibitor of type I collagen synthesis, attenuates osteoarthritis by inhibition of TGF-β activity and type H vessels in subchondral bone (Cui et al., 2016).

In acute myeloid leukemia (AML), type H capillaries are reduced in endosteum vascular niche (Duarte et al., 2018). Chemio- and radio-therapy in AML induce an increase in type H vessels, leading to an expansion of pericytes and cancer stem cell quiescence (Singh et al., 2019).

## 6 Concluding remarks

Maintaining bone mass and homeostasis depends on the intimate relationship between angiogenesis and osteogenesis (Gerber and Ferrara, 2000). To control skeletal remodeling and repair, endothelial cells produce angiocrine factors, while osteogenic cells produce angiogenic factors. Another illustration of the significant role that organ-specific endothelial cells play in the regulation of vascular niche functions during organ development as well as under various physiological and pathological conditions, such as inflammation, aging, cancer, and metastasis. These conditions include regeneration and homeostasis. In this context, blood vessels play crucial roles in illness development as well as disease control and are important targets for cutting-edge therapeutics.
